# Evaluation of Serum Levels of Inflammation, Fibrinolysis and Oxidative Stress Markers in Coronary Artery Disease Prediction: A Cross-Sectional Study

**DOI:** 10.5935/abc.20190214

**Published:** 2019-10

**Authors:** Iran Castro, Hugo Fontana Filho

**Affiliations:** 1 Fundação Universitária de Cardiologia - Instituto de Cardiologia (ICFUC), Porto Alegre, RS - Brazil

**Keywords:** Coronary Artery Diseases/physiopathology, Biomarkers, Inflammation, Oxidative Stress, Fibrinolysis

When assessing patients with coronary artery disease (CAD) traditional risk factors such as diabetes mellitus, hypertension, dyslipidemia, smoking, stress, physical inactivity, obesity, and family history are not found in 20% of the patients,^1^ and up to 40% of patients have only 1 risk factor.^2^ Considering the high prevalence of CAD and the fact that despite the numerous efforts made in primary prevention, the disease still has a high incidence, identifying markers that can predict at-risk patients is a goal that should always be pursued.

However, when evaluating a possible marker of disease, it must meet certain criteria. It should identify individuals at risk (accuracy), its results should be the same when repeated in other patients (reliability) and, especially, it should allow early intervention aiming at reducing the incidence of the problem (therapeutic impact).^3^

The discovery and validation model of a biomarker first comprises its detection, followed by its evaluation in patients with and without the disease. Afterwards, retrospective studies are analyzed to determine whether there is a threshold that differentiates cases and controls to detect the test positivity threshold. Subsequently, screening tests are prospectively applied to large cohorts. Finally, the biomarker is validated in a randomized clinical trial.^4^

The present study,^5^ using a cross-sectional design, evaluated serum levels of inflammation, fibrinolysis and oxidative stress markers in 4 groups of patients with suspected CAD (3 of them with different degrees of CAD and 1 group without lesions) and 1 control group. The analysis showed that serum levels of high sensitivity C-reactive protein (hs-CRP), sialic acid, vitronectin, plasminogen-1 activator inhibitor, and oxidized low-density lipoprotein (Ox-LDL) were significantly higher in the CAD groups than in the control group. As expected, smoking, hypertension, and diabetes were more prevalent in the CAD group than in the control group, showing that traditional risk factors are likely to be associated with increased inflammatory, fibrinolysis, and oxidative stress levels. The evaluation of the levels of CAD markers was not studied prospectively, and it is impossible to assess whether the reduction in serum levels would be related to a better prognosis.

To date, the only marker that meets all the aforementioned criteria, i.e., accuracy, reliability and therapeutic impact, seems to be the hs-CRP. Primary prevention studies using statins showed a decrease in outcomes and markers after intervention in an apparently healthy group.^6^ Secondary prevention studies using monoclonal antibodies (canakinumab) that reduce inflammatory activity have also reduced events, irrespective of LDL levels,^7^ raising the possibility that in the near future the much desired reduction in the so-called residual risk may be an attainable target.
